# A Clinical Suspicion of Quetiapine-Induced Psychosis: A Case Report and Literature Review

**DOI:** 10.7759/cureus.52167

**Published:** 2024-01-12

**Authors:** Michael J Valentine, Ankur Kayastha, Takara R Newsome-Cuby, Anh Thu N Nguyen, Riley G Fisher, Hanh M Pham, Saif A Meimon, Alexander Phu, Connor A Parry, Joshua J Nelson, Ethan C Hayes, Sunita Muranjan

**Affiliations:** 1 College of Osteopathic Medicine, Kansas City University, Kansas City, USA; 2 College of Life Sciences, Brigham Young University, Provo, USA; 3 Public Health, University of Nebraska Medical Center, Omaha, USA; 4 College of Osteopathic Medicine, Kansas City University, Joplin, USA; 5 Psychiatry, Integrated Psychiatric Consultants, Kansas City, USA

**Keywords:** naranjo score, naranjo scale, seroquel, brief psychotic disorder, substance-induced psychosis, hallucinations, ziprasidone, drug-induced psychosis, psychosis, quetiapine

## Abstract

Quetiapine, a pharmacological agent within the class of atypical antipsychotics, is characterized by its efficacy in mood stabilization and its role in the modulation of serotonergic and dopaminergic pathways. Its therapeutic utility is broad, encompassing the management of acute psychotic episodes, schizophrenia, bipolar disorder, and treatment-resistant depressive states. Quetiapine's effectiveness extends to depressive disorders that do not exhibit classic psychotic features, with a side effect profile that is less burdensome than many alternative psychotropic medications. Its versatility in addressing a range of psychiatric conditions is useful in the psychopharmacological management of mood and thought disorders. However, like all drugs, quetiapine may have different effects relative to the individual. It is imperative to approach the administration of quetiapine carefully, ensuring any adverse effects are ameliorated for beneficial therapeutic outcomes. In this case report, we present a psychosis-naive 42-year-old male who developed psychotic symptoms after beginning a quetiapine regimen in order to manage major depressive disorder with suicidal ideation. Clinical suspicion of quetiapine-induced psychosis was a diagnosis considered due to symptom remission secondary to ziprasidone in the place of quetiapine. The determination of a suspected adverse drug reaction can utilize the Naranjo scale to demonstrate the likelihood of an adverse drug reaction. This patient scored a three on the Naranjo scale, indicating a possible adverse effect from quetiapine. Other potential etiologies of psychosis include medication-induced psychosis, major depressive disorder exacerbation, cocaine use/withdrawal, and brief psychotic disorder. Quetiapine-induced psychosis has not been described in the current literature, and therefore, this case report is solely based on clinical evaluation and is intended for educational purposes due to possible confounding factors and etiologies.

## Introduction

Quetiapine is categorized within the class of second-generation antipsychotics (SGAs), also known as atypical antipsychotics. These agents have a mechanism of action that primarily antagonizes dopamine 2 (D2) and serotonin 2A (5-HT2A) receptors. However, quetiapine, in particular, has a higher affinity for 5-HT2A receptors. Quetiapine is selective for the mesolimbic and mesocortical dopamine systems [1]. Significant risks are noted in the form of black box warnings, which highlight an increased risk of suicidal behavior and mortality rates in the elderly who have dementia-related psychopathology [2]. These concerns also include a significant risk for suicidal ideation in persons under 65 years of age and an increased incidence of stroke and mortality in the geriatric population exceeding this age threshold [3].

Quetiapine's therapeutic applications are not limited to antipsychotic indications despite its pharmacological class name. It also functions effectively as a monotherapy or as a supplementary agent in the treatment of refractory major depressive disorder (MDD) [4]. The drug's spectrum of potential side effects includes antimuscarinic, antiadrenergic, antihistaminic, and antidopaminergic adverse reactions. Symptoms may include weight gain, dry mouth, nausea, lethargy, and muscle stiffness. Elevated serum glucose and lipid levels may also be observed [5]. Notably, SGAs also carry a higher risk for the development of neuroleptic malignant syndrome (NMS) [6], a rare but potentially life-threatening reaction. Therefore, the administration of quetiapine should be approached with expectant management, close observation, and patient-specific considerations.

In recent years, depression and suicidal ideation have witnessed an alarming surge on a global scale. Over one-third of American women report having been diagnosed with depression at some point in their lives, compared to an estimated 20% of American men [7]. A parallel trend has been observed in a study encompassing 27 European nations, indicating a higher prevalence of depression among women [8]. While the etiological factors remain complex and multidimensional, there is considerable evidence suggesting a genetic predisposition to depression [9]. Genetic factors appear to strongly influence earlier manifestation of depressive symptoms within younger demographics [9]. Another prevalent theory asserts that the increasing incidence of depression may be attributable to increased screen time and technological engagement [10,11].

Psychosis, on the other hand, is conceptualized as a detachment from reality rather than a discrete medical condition. For diagnostic and clinical management, psychosis is considered a cluster of symptoms that most prominently include auditory/visual hallucinations and delusions. Hallucinations are sensory perceptions that occur without an external stimulus, such as auditory hallucinations wherein the individual hears voices without external sound. Delusions are unfounded beliefs that an individual unreasonably and tenaciously holds [12,13]. Psychosis can emerge from an underlying psychiatric condition such as schizophrenia or as an atypical exacerbation of certain mood disorders like bipolar disorder and MDD [14]. Additional etiologies include dementia, central nervous system infections, nutrient deficiencies, and substance use. 

Quetiapine, an effective SGA with a relatively benign side effect profile, is commonly used to manage depression and psychosis. It is highly unusual for this antipsychotic to induce psychiatric symptoms [1]. In this case report, we present a 42-year-old male who seemingly developed psychotic symptoms following a regiment of quetiapine in an acute inpatient psychiatric care setting. A literature review was also conducted on other possible etiologies that may have resulted in this presentation.

## Case presentation

A 42-year-old male presented voluntarily to an acute inpatient psychiatric hospital with worsening depression and suicidal ideation with a plan. He reported intrusive thoughts of hurting himself. He is married and has a young child. Past psychiatric history includes previous suicidal ideation, MDD, and general anxiety disorder (GAD). Past medical history includes hypertension. He denied alcohol and tobacco use but admitted to recreational cocaine use once a month for the past five years. Medications include bupropion (300 mg daily for MDD), escitalopram (10 mg daily for MDD and GAD), and alprazolam (0.25 mg up to three times a day as needed for breakthrough anxiety). 

Vital signs include a blood pressure of 134/85 mmHg, a temperature of 97.7 degrees Fahrenheit, a heart rate of 65 beats per minute (BPM), and a respiratory rate of 16. He scored a 22 on the Columbia Suicide Severity Rating (C-SSR) scale. On general examination, he avoided eye contact, had overgrown facial hair, and communicated with muffled speech. His mood was depressed, congruent with dysthymic affect. Notably, he reported no history of hallucinations or delusions. A mental status exam revealed his judgment as poor, speech as tangential, memory as fair, and alert and oriented to person, place, time, and event. 

Considering the beneficial environment of close monitoring within an inpatient unit and refractory psychiatric conditions, the patient was initiated on a regimen of quetiapine (100 mg daily) to address his worsening anxiety symptoms, MDD, and suicidal ideation. Following a five-day period of comprehensive treatment that included psychotherapy and group therapeutic activities, there was a significant improvement in his mental status examination, with depression levels self-reported at 2/10. Subsequently, the patient presented to the multidisciplinary healthcare team for a comprehensive review to evaluate his suitability for discharge. He met the established criteria, verbalized stability on his medication regimen, and expressed an intent to transition to a residential treatment facility after discharge from the acute inpatient psychiatric setting. The psychiatric team reached a consensus that he was an appropriate candidate for discharge.

However, a critical event unfolded on the sixth night of admission. The patient’s roommate reported that the patient was experiencing hallucinations, evidenced by conversational engagement with the walls and muttering to himself throughout the night. By morning, his behavior escalated to a heightened level of agitation. His roommate reported that the patient did not have any sleep that night. Clinical staff directly observed him frantically traversing the hallways and rummaging under furniture for what he described as looking for a message. He also aggressively searched the pockets of a social worker, ostensibly in search of this hidden message.

Physiological indicators were abnormal: bilaterally dilated pupils and tachycardia with a pulse rate of 105 beats per minute. Other vital signs were unattainable due to agitation. The pharmacologic intervention was immediately administered in the form of a “B52 cocktail” comprising lorazepam (2 mg), haloperidol (10 mg), and diphenhydramine (50 mg) which induced sleep but did not abate psychosis. He was given additional B52 cocktails over the next two days. In light of these developments, quetiapine was discontinued on the suspicion that it may have precipitated psychotic symptoms. All other medications were maintained, including his bupropion. Ziprasidone (20 mg) was instituted as a temporary replacement. The patient was able to sleep that night. His hallucinations completely ceased on the ninth day of admission, and his mental state stabilized. Ziprasidone was then prescribed as a replacement for quetiapine.

The administration of a urine drug screen test ruled out external drugs. The results were negative for all drugs except for benzodiazepines, which was expected due to lorazepam included in the B52 cocktail. Considering the controlled environment, it seemed unlikely that other patients smuggled in recreational drugs. However, it is not impossible. Historical records from the inpatient unit reveal that there have been incidents of patients smuggling contraband into the acute care setting when the unit was under previous healthcare ownership. Upon investigation, there was no evidence of contraband smuggling. In addition, the patient had repeatedly denied taking other patient medications. Quetiapine-induced psychosis was a diagnosis formulated from clinical suspicion upon the ruling out of other etiologies, although it is possible that the patient may have had a psychotic episode secondary to environmental triggers, MDD, medication use, cocaine use/withdrawal, or unknown etiology. There is no record of other precipitating events or past medical history of psychosis. Following close surveillance for five days (14th day of admission) with ziprasidone, goal attainment and self-reported amelioration of depression symptoms allowed him to transition to a long-term residential treatment facility.

## Discussion

Between the years 2010 and 2018, there was a noteworthy escalation in the number of American adults receiving a diagnosis of MDD, rising from 15.5 million to 17.5 million individuals [15]. The 12-month prevalence of medication-treated MDD in the United States stood at an estimated 8.9 million adults [16]. The economic ramifications of this growing incidence manifested in a surge from $US 236.6 billion in 2010 to $326.2 billion by 2020 [15]. Of this population, approximately 31% or 2.8 million individuals were categorized as treatment-resistant [16]. Collectively, these statistics demonstrate a critical gap in American psychiatry that illuminates a need for targeted intervention.

Many American acute psychiatric units are grappling with a chronic, multifaceted crisis that has long been an issue of concern within the medical community [17,18]. This problem is postulated to be a byproduct of insufficient treatment resources and loss of follow-up, leading to a cyclical pattern of readmissions [17]. Compounding this challenge is the considerable economic burden of inpatient psychiatric care [15,18,19]. It is a widely acknowledged principle among psychiatric professionals that the successful management of psychiatric disorders extends far beyond pharmacological interventions alone. In this case, our medical team and staff embody a biopsychosocial management viewpoint. This perspective posits that effective treatment of psychotic episodes requires a multidisciplinary approach inclusive of various healthcare providers beyond medical management. Social workers and psychologists play an integral role in crafting a well-rounded psychiatric care plan. Focusing on only one aspect of this biopsychosocial triad is an imbalanced approach that is not only therapeutically suboptimal but may also increase the likelihood of patient relapse. 

It is also well known that individuals diagnosed with depression are more frequently subject to polypharmacy [20], a trend that is amplified within the context of acute inpatient psychiatric care. In these settings, healthcare providers may find themselves increasingly reliant on potent antipsychotic medications, especially when confronted with cases that are resistant to abortive measures. On the one hand, the inpatient setting facilitates the prompt deployment of pharmacological interventions with active management, but on the other, it risks fostering an overreliance on medication.

In this case, the patient presented with treatment-resistant depression, pronounced anxiety, and persistent suicidal ideation, all of which collectively led to the clinical decision to consider the use of antipsychotics as an adjunctive treatment modality. His status as an inpatient in a highly regulated setting provided a beneficial environment to initiate and evaluate the effects of antipsychotic therapy. Quetiapine was administered at a dosage of 100 mg in the evening, with the patient undergoing close observation at 15-minute intervals. Daily follow-ups were conducted to monitor his tolerance to the medication. Initially, direct evaluations revealed that the patient remained alert and oriented with no hallucinatory behavior, allowing the quetiapine treatment to be continued under stringent monitoring.

It was on the third day of quetiapine therapy that the patient began to exhibit clear signs of hallucinations, delusions, and disorganized thinking and speech. According to DSM-5 criteria, his condition would be classified as a brief psychotic disorder characterized by the presence of psychotic symptoms lasting between one day and one month. Furthermore, it remains possible that his psychotic episode developed from other undiagnosed etiologies.

Given the absence of any prior psychotic events in the patient's history, a psychiatric cause for his psychosis was considered unlikely. Behavioral changes of an acute nature are more frequently observed in the geriatric population and often manifest as delirium [21]. However, the possibility of delirium was discounted in this instance as the administration of a benzodiazepine enabled the patient to sleep, yet his psychotic state persisted into the following day. Notably, this patient is not a geriatric patient. It should be acknowledged that numerous medical conditions have been recognized as potential inducers of psychosis [22]. Exhaustive cataloging of such conditions is beyond the scope of this report, but a selection of pertinent medical etiologies can be reviewed in Table 1. Undiagnosed medical conditions as a cause of this patient’s psychosis cannot be ruled out. Furthermore, unknown environmental triggers encountered by the patient could have also exacerbated this psychotic episode.

**Table 1:** A brief summary of medical conditions that may induce psychosis. [22-25]

Psychosis: Potential Causes	Psychosis: Potential Pathologies
Dementia	Lewy body dementia, Alzheimer's disease
Demyelinating Diseases	Multiple sclerosis, leukodystrophies
Neuropsychiatric Disorders	Huntington disease, Wilson disease, Parkinson dementia
Autoimmune Disorders	Systemic lupus erythematosus, Autoimmune encephalitis
Infections	Viral encephalitis, Neurosyphilis, Lyme disease, Neurocysticercosis, Sarcoidosis, Prion disease
Nutritional Deficiencies	Vitamin A, Vitamin D, zinc, Niacin, Vitamin B12, Magnesium

Our patient, who has a history of cocaine addiction, was under close observation. Despite the stringent controls typically exercised within acute care settings, the possibility of medication access through other patients or unauthorized drug introduction during visitation hours cannot be excluded. Protocols on this unit require that patients ingest their medications under nursing supervision, demonstrating that they have swallowed their doses. An exhaustive interview process yielded consistent denials from the patient regarding the consumption of another individual’s medication. Additionally, a toxicology screen conducted on the patient returned negative results for illicit drugs, with the notable exception of benzodiazepines, which were administered to address his psychotic symptoms. Substances used recreationally are frequently implicated in the onset of psychotic episodes [22-25]. An exhaustive list is outside the scope of this case report. However, a condensed list of such substances can be found in Table 2. Recent research involving Rhesus monkeys has suggested that quetiapine, typically devoid of abuse potential, may exacerbate cocaine-seeking behaviors at low doses when addiction is already present [26]. Consequently, Brutcher et al. recommend vigilant monitoring of quetiapine in patients with a history of cocaine dependence [27].

**Table 2:** A brief summary of recreational drugs that may induce psychosis. [22-25]

Substance	Recreational Substance-Induced Psychosis Example
Alcohol	Alcohol toxicity and withdrawal
Stimulants	Methamphetamine, Dextroamphetamine, Cocaine
Depressants	Anticholinergics (Antimuscarinics), Antihistamines, Antihypertensives, Antiepileptics
Hallucinogens	Ecstasy, Phencyclidine (PCP), Lysergic acid diethylamide (LSD)

Quetiapine is consistently recognized for its efficacy in the management of psychotic episodes [1,3]. While it may seem paradoxical for an antipsychotic medication to contribute to psychosis, the cessation of quetiapine and initiation of ziprasidone corresponded to the resolution of psychotic symptoms in the patient. It is important to emphasize that the patient maintained his normal medication regime outside of the change from quetiapine to ziprasidone, making the likelihood of bupropion-induced psychosis unlikely. 

Due to the lack of concrete evidence, the Naranjo Adverse Drug Reaction Probability Scale was employed to determine the probability of an adverse drug reaction. The Naranjo Algorithm is a clinical worksheet that is useful for demonstrating the probability of adverse drug reactions [28]. This worksheet contains 10 questions with three answer responses that are scored. The higher the score, the more likely the adverse reaction is due to the implicated drug. In this case, our patient scored a three on the Naranjo Algorithm indicating a possible adverse drug reaction. The scoring of the Naranjo worksheet is depicted in Table 3 and Table 4.

**Table 3:** The Naranjo Algorithm is used for assessing the probability of adverse drug reactions. A score of 9 or more is considered to be a definite adverse drug reaction. The patient scored a three on the algorithm, demonstrating a possible adverse drug reaction. [28]

Question	Yes	No	Do Not Know	Score
1. Are there previous conclusive reports on this reaction?	1	0	0	0
2. Did the adverse event appear after the suspected drug was administered?	2	-1	0	2
3. Did the adverse event improve when the drug was discontinued or a specific antagonist was administered?	1	0	0	1
4. Did the adverse event reappear when the drug was readministered?	2	-1	0	0
5. Are there alternative causes that could on their own have caused the reaction?	-1	2	0	-1
6. Did the reaction reappear when a placebo was given?	-1	1	0	0
7. Was the drug detected in blood or other fluids in concentrations known to be toxic?	1	0	0	0
8. Was the reaction more severe when the dose was increased or less severe when the dose was decreased?	1	0	0	0
9. Did the patient have a similar reaction to the same or similar drugs in any previous exposure?	1	0	0	0
10. Was the adverse event confirmed by any objective evidence?	1	0	0	1
	Total Score: 3			

**Table 4:** Scoring interpretation for the Naranjo Adverse Drug Reaction Probability Scale. [28]

Score	Interpretation of Scores
Total Score: ≥9	Definite. The reaction (1) followed a reasonable temporal sequence after a drug or in which a toxic drug level had been established in body fluids or tissues, (2) followed a recognized response to the suspected drug, and (3) was confirmed by improvement on withdrawing the drug and reappeared on re-exposure.
Total Score: 5 to 8	Probable. The reaction (1) followed a reasonable temporal sequence after a drug, (2) followed a recognized response to the suspected drug, (3) was confirmed by withdrawal but not by exposure to the drug, and (4) could not be reasonably explained by the known characteristics of the patient’s clinical state.
Total Score: 1 to 4	Possible. The reaction (1) followed a temporal sequence after a drug, (2) possibly followed a recognized pattern to the suspected drug, and (3) could be explained by characteristics of the patient’s disease.
Total Score: ≤0	Doubtful. The reaction was likely related to factors other than a drug.

It remains clinically plausible that the psychiatric symptoms experienced by the patient may not have been optimally managed with quetiapine. In other words, an undiagnosed psychiatric disorder may have been exacerbated in the acute inpatient setting and was not well-controlled by the prescribed quetiapine regimen. MDD itself may also be responsible for the emergence of psychotic features [29]. Such features have been reported to have a prevalence of approximately 11% (out of 1410 patients) [14]. 

Given the complexities in psychiatric symptoms, diagnostic classification, and individual drug responses, the various causes of psychosis secondary to MDD become blurred. Antipsychotic medications generally represent a cornerstone in the management of such conditions, but individual variation in therapeutic response warrants a patient-centered approach. Nonetheless, psychosis treatment is contingent upon ensuring patient safety. If psychosis severity increases or is not reasonably ameliorated after administration of antipsychotics, such as in the case of this patient with quetiapine, then it is paramount to discontinue the antipsychotic and consider an alternative medication.

## Conclusions

The occurrence of quetiapine-induced psychosis is rare, considering quetiapine's reputation for mitigating psychosis. In a well-documented instance, a patient with no prior history of psychosis was initiated on a quetiapine regimen that precipitated psychotic behavior within three days of treatment. In addition to delusions and hallucinations, this patient exhibited erratic behavior and abnormal physiologic indicators (tachycardia and dilated pupils) resembling a psychotic episode. The administered toxicology screen returned negative for any illicit substances, with the notable exception of benzodiazepines (from B52 cocktail sedation management). The patient scored a three on the Naranjo Adverse Drug Reaction Probability Scale, indicating a possible adverse drug reaction. Notably, the cessation of quetiapine was followed by remission of psychotic symptoms, leading to the clinical suspicion of quetiapine-induced psychosis. Other etiologies such as brief psychotic disorder, bupropion-induced psychosis, cocaine use/withdrawal, MDD exacerbation, and unknown origin are also plausible. Antipsychotic medications generally represent a cornerstone in the management of such conditions, but individual variation in therapeutic response warrants a patient-centered approach. Despite potential confounds in the etiology of psychotic symptoms in this case, treatment is contingent upon ensuring patient safety. If psychosis severity increases or is not reasonably ameliorated after administration of antipsychotics, then it is paramount to discontinue the antipsychotic and consider an alternative medication.
